# strum: an R package for *stru*ctural *m*odeling of latent variables for general pedigrees

**DOI:** 10.1186/s12863-015-0190-3

**Published:** 2015-04-09

**Authors:** Yeunjoo E Song, Catherine M Stein, Nathan J Morris

**Affiliations:** Department of Epidemiology and Biostatistics, Case Western Reserve University, Cleveland, OH 44106 USA; Center for Proteomics and Bioinformatics, Case Western Reserve University, Cleveland, OH 44106 USA; Center for Clinical Investigation, Case Western Reserve University, Cleveland, OH 44106 USA

**Keywords:** Structural equation modeling, Latent variable analysis, Pedigree data, Genetics, Genetic epidemiology, Simulation, Visualization

## Abstract

**Background:**

Structural equation modeling (SEM) is an extremely general and powerful approach to account for measurement error and causal pathways when analyzing data, and it has been used in wide range of applied sciences. There are many commercial and freely available software packages for SEM. However, it is difficult to use any of the packages to analyze *general* pedigree data, and SEM packages for genetics are limited in their application.

**Results:**

We present the new R package *strum* to serve the need of a suitable SEM software tool for genetic analysis. It implements a general framework for SEM within the context of general pedigree data. This context requires specialized considerations such as familial correlations and ascertainment. Our package is an extraordinarily flexible tool capable of modeling genetic association, linkage analysis, polygenic effects, shared environment, and ascertainment combined with confirmatory factor analysis and general SEM. It also provides a convenient tool for model visualization, and integrates tools for simulating pedigree data. The various features of this package are tested through a simulation study to evaluate performance, and our results show that *strum* is very reliable and robust in terms of the accuracy and coverage of parameter estimates.

**Conclusions:**

*strum* is a valuable new tool for genetic analysis. It can be easily used with general pedigree data, incorporating both measurement and structural models, giving it some significant advantages over other software packages. It also includes a built-in approach for handling ascertainment, a helpful integrated tool for genetic data simulation, and built-in tools for model visualization, providing a significant addition to biomedical research.

## Background

SEM is an extremely general and powerful multivariate analysis approach used to estimate a system of linear equations to test the fit of a hypothesized “causal” model and account for measurement error when analyzing data [1]. This is accomplished by estimating the parameters for a system of simultaneous equations that are developed based on a hypothesized model, and varying the models to identify a most parsimonious and best fitting model. SEMs comprise two sub-models. The measurement model evaluates latent variables using observed variables, also referred to as indicators; this is the same framework used in factor analysis. The structural model then estimates the relationships between the latent variables as well as other observed variables. Though SEM has been largely utilized in the social sciences [1], it also has utility for genetic epidemiology. In many studies of complex genetic traits, several correlated phenotypes are measured, some of which may have causal relationships amongst themselves. SEM may be used to disentangle these causal relationships, and also evaluate the independent influences of multiple genetic variants in these complex networks of phenotypes, longitudinal models, environmental factors, and other covariates [2].

As SEM has been used in wide range of applied sciences, there are many commercial and freely available software packages for SEM [2,3]. To name a few, LISREL [4], EQS [5], AMOS [6], CALIS (in SAS) [7], SEPATH (in Statistica) [8], Mplus [9], and Mx [10] are the popular choices. There are also packages available in R [11]: lavaan [12], sem [13], lava [14], and OpenMx [15]. Narayanan [3] provided a comprehensive review of eight different packages for a variety of criteria from documentation to parameter estimation. As pointed out by Narayanan [3], different options are available in each package, and the special features in each package make users of SEM choose one package over the rest for their particular needs.

It is possible to do genetic analysis using some of the SEM packages above, especially for twin data and nuclear family data [16]. However, as we and others reviewed elsewhere [2,3], the packages do not natively allow the estimation of both the measurement model and structural model simultaneously with estimation of polygenic effects, linkage and association effects, and ascertainment correction in generalized pedigrees. It should be understood that the correlation structures used in a polygenic model or linkage model are more complex than simply adding a random effect for each pedigree. The polygenic model very specifically accounts for the fact that the phenotypes of more closely related individuals will be more strongly correlated than the phenotypes of distantly related individuals. This can become complicated with general pedigree structures such as: variable family sizes, multiple generations, inclusion of extended relationships such as half-siblings and cousins and pedigree loops. Dealing with ascertainment (i.e. the selection of pedigrees based on the extreme phenotype of an index case) is of particular importance in pedigree data. While it may be possible to trick a general purpose multilevel SEM software package into performing some of these tasks, our package interacts with general pedigree structures natively, and in a very natural way. The lack of a suitable SEM software tool for genetic analysis has also been discussed elsewhere [2].

For the visualization of an SEM, some packages generate a dot file to be used in a tool to lay out the model graphically [13-15], while other programs incorporate a graphical user interface, so the user can draw the model interactively [4-6,17]. A bridge package semPlot is another choice for the visualization only [18], and the program psych has functions for graphical display of SEM [19]. However, most tools lack the flexibility to draw more than one variance component in the model, which is common in genetic analysis. Also, few tools provide the visualization as the convenient built-in component with a function call.

Previously, we developed a robust and flexible framework for SEM in general pedigree data. It not only can handle both measurement and structural models, but it can also estimate polygenic variance effects, genetic linkage effects and association effects while correcting for ascertainment, which sets it apart from other SEM methods for genetics [20]. One of the primary conceptual innovations of this framework is that it enables the analyst to mentally separate the model of familial correlation from the causal/measurement model by the use of Kronecker notation. Here, we present *strum*, a user-friendly R package that implements this framework, and also provides visualization, which is not a common built-in component of most SEM R packages.

## Implementation

The statistical details of the framework implemented by *strum* are described by Morris et al. [20], and creating the R package involved a considerable amount of additional innovative work. Briefly, our method uses Kronecker product notation to model the covariance among relatives as well as the covariance among measured variables (phenotypes, genes, covariates, etc.), and allows for the estimation of polygenic effects, linkage and/or association effects, and incorporates an ascertainment correction. For a flavor of the theory behind our modeling approach, consider fitting a polygenic model with *t* observed traits. Let **y**_k_ represent the vector all the traits for each individual stacked on top of each other for the *k*^th^ pedigree, **Φ**_k_ represent the kinship coefficient matrix for the *k*^th^ pedigree and **I**_k_ represent the identity matrix with dimensions equal to the size of the *k*^th^ pedigree. Also, let **V**_*p*_ (**θ**) and **V**_*e*_ (**θ**) represent *t × t* variance covariance matrices for the polygenic and environmental effects written as functions of some model parameters (**θ**). We assume that the covariance of **y**_k_ may be written as var (**y**_k_) = **Φ**_*k*_ ⊗ **V**_*p*_ (**θ**) + **I**_*k*_ ⊗ **V**_*e*_ (**θ**), where ⊗ is the Kronecker product. The use of such a polygenic covariance structure for pedigree data allows the analyst to focus on specifying a set of equations and latent variables to model **V**_*p*_ (**θ**) and **V**_*e*_ (**θ**). The model syntax allows the analyst to easily include or exclude polygenic random effects from both observed and latent traits.

Before model fitting, models must first be algorithmically parsed into a set of matrices which are functions of the model parameters. As described elsewhere [20], the model fitting process then proceeds in two stages. In the first stage, the “saturated” model parameters are estimated by using maximum likelihood to fit a univariate variance component model for each variable and then to fit a bivariate variance component model for each pair of variables. In the second stage, *strum* seeks the parameters which minimize the distance between (a) the observed mean and variance components (as found in stage 1) and (b) the mean and variance components implied by the parameters. We do not force the variances to be positive because this can create unpredictable asymptotic distributions as we have shown elsewhere [21]. A robust sandwich type estimator is used to estimate the standard errors of the parameter estimates [22-24]. The sandwich type estimator makes the standard errors and p-values asymptotically valid in terms of coverage and type 1 error rate even in the presence of data that is not multivariate normally distributed. However, data that is not multivariate normal may reduce the efficiency and the power. As with all SEM software, there is always a danger that the estimates are based on a local optima. To help avoid local optima, *strum* generates multiple random starting values via an innovative heuristic genetic/evolutionary algorithm. Much of the internal code is written in C++ for computational efficiency.

The chi-squared test of model fit is a commonly used test of the null hypothesis that the model fits the data well. The *strum* package presents several different versions of the chi-square test correcting for first or second moments as discussed by Morris et al. [20]. In addition, *strum* implements what we refer to as the “theoretically corrected” chi-square which is based on the true asymptotic distribution of the distance calculated in stage 2 of the fitting process. The true asymptotic distribution is the same distribution as a weighted sum of chi-squared random variables with weights determined using Eigen decomposition. See for example equation 2 of Satorra and Bentler [25]. To determine the p-value, we simulate from the asymptotic distribution using the estimated weights. We then back calculate a chi-square statistic which would have produced the p-value. Comparative Fit Index (CFI) using this theoretically corrected chi-square and the degree of freedom is reported as well.

The *strum* package has an intuitive interface for inputting and fitting models using a set of simple operators which are parsed by the package. It includes both fitting and simulation of a broad range of models including latent measurement models, structural equation models with covariates, and including latent growth curve models. It can also handle multilevel models, polygenic random effects and linkage random effects. The syntax is very similar to that employed by lavaan [12], so that the development of measurement and structural equations is intuitive and elegant. Also, being implemented as a package in R [11], which is a commonly used and well documented software tool, adds more value into the *strum* package. The following section lists features that make *strum* unique.

### Pedigree data

The *strum* package can be used for general pedigree data with many different types of relative pairs as well as individual data, including pedigrees with loops. Pedigree size, the number of pedigrees, and presence of loops is not restricted by strum, but may be limited by the system (eg. Memory size, CPU speed etc.). Large pedigrees with many traits may make the make the estimation process slow. The general framework allows the analyst to easily separate the model for familial correlation from the structural part of the model. This makes it simple to incorporate polygenic effects, common environmental effects (e.g. household effects) and linkage effects into the SEM. Thus *strum* is, in some ways, a generalization of variance component based methods/software for general pedigrees [26].

### Genetic analysis

The *strum* package can be used for a broad array of genetic analysis within SEM. It allows many different types of statistical analyses to be incorporated including genetic association and linkage analysis. Other types of analysis models can be done easily, like SEM with latent variables with a polygenic effect, confirmatory factor analysis (CFA) of a pleiotropic genetic effect influenced by a SNP, multiple SNP latent genotype models, Mendelian randomization models, and the classic “ACE model” that estimates components of variance due to additive genetic (A), shared environment (C), and residual environment (E) [27]. Additionally, *strum* can estimate polygenic variance and covariances among variables. Again, the general framework of *strum* allows these analyses to be done easily and combined in creative and unique ways.

### Ascertainment

One obvious issue that arises with pedigree data analysis is ascertainment bias because the families are not selected at random but through one or more affected individuals, called probands. Ascertainment in pedigree analysis is a difficult issue which has been discussed by many researchers [28]. The *strum* package implements an approach that uses the likelihood conditional on the actual trait value of the proband, assuming single ascertainment [20].

### Simulation

The *strum* package can simulate multivariate pedigree data making it an ideal tool to understand how well different methods work. The simulation integrates with HapMap data [29] to create realistic patterns of linkage disequilibrium for the pedigree founders. Given a pedigree structure as input, it performs gene dropping to the offspring. It also simulates polygenic correlations between family members and genetic associations within the overall SEM context. Ascertainment can also be simulated.

### Visualization

A major addition since our 2010 work [20], the *strum* package includes a “built-in” tool for model visualization, which few SEM programs have. Unlike some of the other SEM tools introduced previously, the visualization of models in *strum* is done by a simple native R function call, plot() with the model name. It can plot the classic path diagram of the model utilizing the existing R package Rgraphviz [30]. Additionally, it is flexible in its graphical layout types as some models may be more suitable to be laid out other than the classic hierarchical path diagram.

## Results and discussion

The results from a simulation study to evaluate the performance of the *strum* package are reported. We report the biases and mean absolute deviations of model parameters as well as coverage probabilities, i.e., the percentage of replicates with 95% confidence intervals (CI) that covered the true parameter. Additionally, we report the distribution of p-values for the 4 different chi-square tests of model fit. For the ascertainment modeling evaluation, we report the coverage probabilities of 95% confidence intervals for the different settings we tested which demonstrate that our method of ascertainment correction greatly improves coverage rates for ascertained data.

### Models

**Figure 1:**
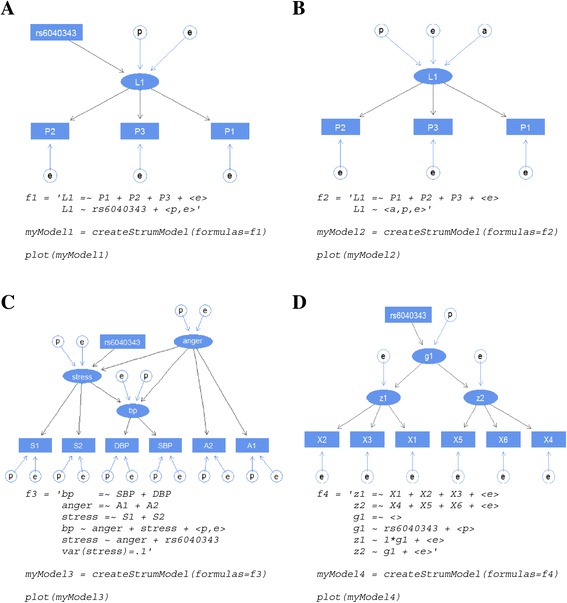
**Analysis models tested in simulations.** The plots and *strum* model formulas are shown for **(A)** Model 1 - Genetic association analysis model with a latent trait, **(B)** Model 2 - Genetic linkage analysis model with a latent trait, **(C)** Model 3 - SEM with multi-level latent variables and polygenic effect, and **(D)** Model 4 - CFA with a pleiotropic genetic effect influenced by a SNP.

We considered 4 different analysis models in the simulation study, trying to cover the different types of analysis that can be done in the *strum* package. For each model, the model diagram plotted using the *strum* package and the R code to construct the model object in the *strum* package is shown in Figure 1. The string value for the formulas argument defines the relationship among variables. The measurement equations are specified by the " = ~" operator. The " ~ " operator specifies the structural equations in the model. The " = " operator specifies the constraints in the model, i.e., fixing a model parameter - a variance, covariance, or coefficient. Please refer to the reference manual for a more detailed description. Figure 1 describes the models which we simulated to evaluate the performance of *strum*.

### Model 1: Genetic association analysis model with a latent trait

Figure 1A shows an example of a typical genetic association analysis model with a latent trait. Suppose that there are three measurements (P1, P2, P3), and it is hypothesized that there is a single latent trait (L1) underlying the three measurements. The latent variable L1 is influenced by a SNP (rs6040343) and a set of variance components, polygenic (p) and random environmental (e). Each trait is also influenced by its own random environmental factor.

### Model 2: Genetic linkage analysis model with a latent trait

Figure 1B shows an example of a typical genetic linkage analysis model with a latent trait using identity by descent (IBD) sharing information. Suppose again that there are three measurements (P1, P2, P3) and a single latent trait (L1) underlying the three measurements. The latent variable L1 is influenced by a set of genetic and random variance components. Each trait is also influenced by its own random environmental factor.

### Model 3: SEM with multi-level latent variables and polygenic effect

Figure 1C is an example of a SEM model with multi-level latent variables and a polygenic effect. Suppose that there are six measurements (A1, A2, SBP, DBP, S1, S2) and three underlying latent variables (anger, bp, stress). anger is measured by (A1, A2), bp measured by (SBP, DBP) and stress is measured by (S1, S2). bp is caused by anger and stress, and stress is caused by anger and a SNP (rs6040343). All traits and latent variables are also influenced by their own polygenic and random variance components except stress, which has the variance fixed at 0.1 for both polygenic and random components.

### Model 4: CFA with a pleiotropic genetic effect influenced by a SNP

Figure 1D is an example of a CFA model with a pleiotropic genetic effect influenced by a SNP. Suppose that there are two sets of three measured traits, which are indicators for two underlying latent variables. The latent variable z1 underlies the first set of three measurements (X1, X2, X3) and the latent variable z2 underlies the second set of three measurements (X4, X5, X6). Both z1 and z2 are influenced by a SNP (rs6040343) through a latent variable g1. Note that the coefficient of g1 to z1 is fixed to be 1. All traits and latent variables are also influenced by their own variance components.

### Simulation

**Figure 2:**
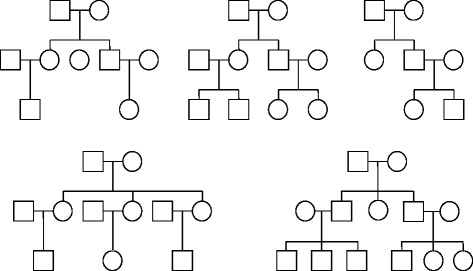
**Pedigree structures used for simulations.**

We simulated two different types of pedigree data. First, we used the pedigree data from the file “example_ped.csv”, which we included in the package, as the base pedigree structures. It consists of 477 individuals from 75 nuclear families with 4 to 11 siblings. The second set of pedigree data consists of 50 individuals from 5 different extended pedigrees with different structures as shown in Figure 2. For the genotypes, we used the chromosome 20 of Hapmap III phased data [29], which is again included in the package. A SNP rs6040343 was selected randomly. Data were simulated under different settings, and for each setting, we simulated 1000 replicates.

In the first simulation design, the overall performance of the 4 different models was assessed. To check the performance under the different sample size, we simulated sets of 75, 150, 300 and 500 pedigrees using two sets of base data above, one with nuclear families only and the other with mixed extended pedigrees. The pedigrees were not ascertained, and the analyses were accordingly done by not modeling the ascertainment scheme. The true value for all parameters equaled 1 for all models. Additionally, for both data sets, we simulated 10000 replicates for Model 1 to assess the type 1 error rate under the null hypothesis of no association between the SNP marker and the latent variable.

The second simulation was designed to evaluate the performance of the ascertainment modeling for Model 3. The pedigrees were ascertained by the simulated values of 6 observed traits in the model. Within a pedigree, a member was assigned to be affected when the mean of 6 trait values was greater than the threshold value of a given prevalence. Among the affected members, the possible proband candidates were randomly selected from the binomial distribution with a given ascertainment probability. To run *strum* analysis without the warning messages for the existence of multiple probands, only the first proband candidate was assigned to be the proband for that pedigree when there existed multiple candidates. Any pedigree without a proband was discarded. The analyses were done with and without modeling the ascertainment. For each type of data set, we again simulated sets of 75, 150, 300 and 500 pedigrees under different prevalence and ascertainment probabilities. We considered 4 different prevalence values – 0.1%, 1%, 5% and 10%. Given these prevalence values, we tested for 3 different ascertainment probabilities, 0.05, 0.1 and 0.2.

### Bias and errors

**Figure 3:**
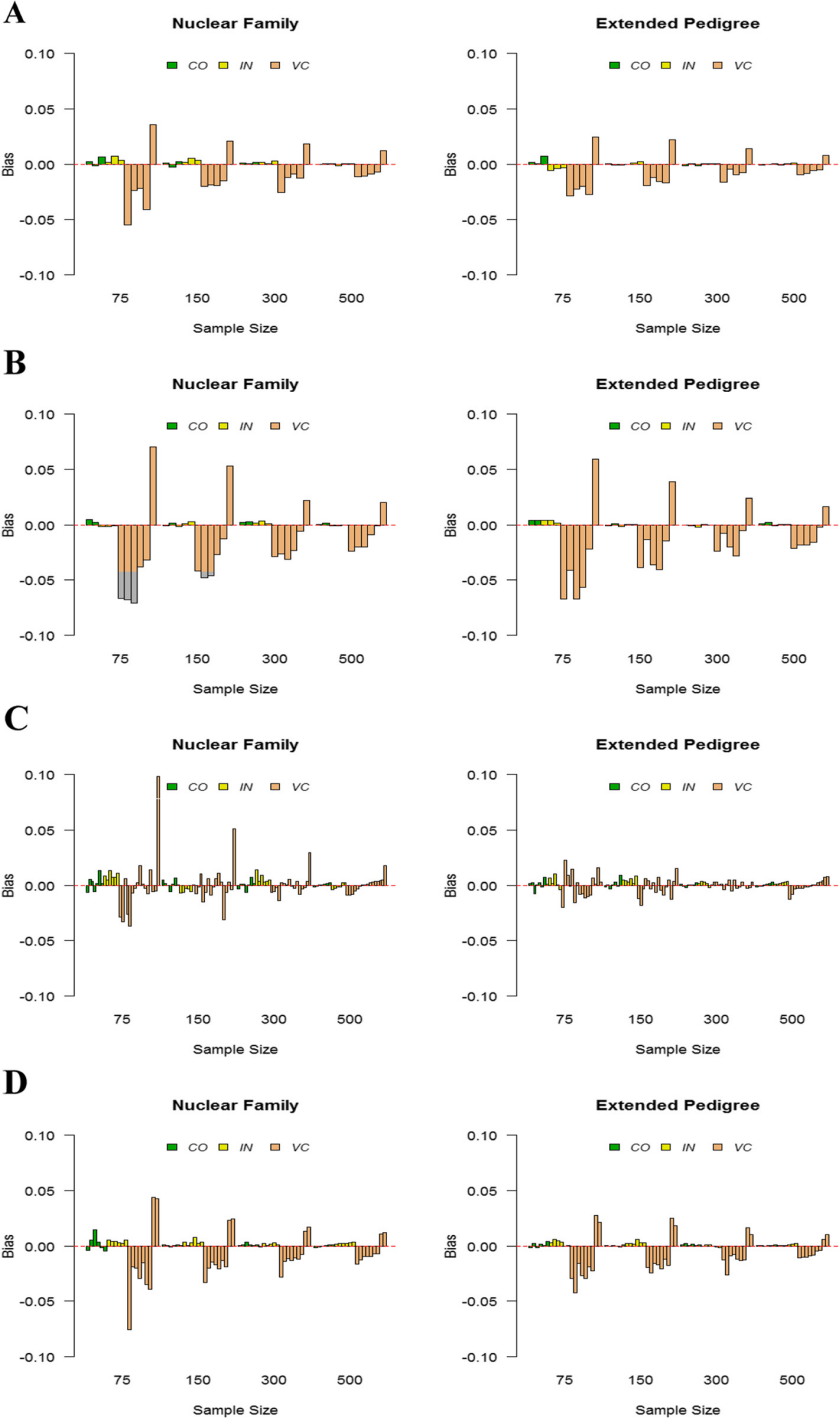
**The bias of each parameter by sample size. A** is for Model 1, **B** is for Model 2, **C** is for Model 3, and **D** is for Model 4 from Figure 1. In each plot, the bars are grouped by three different parameter categories with different colors; green for coefficient parameter (denoted as *CO*), mustard for intercept parameter (denoted as *IN*) and peach for variance component parameter (denoted as *VC*) within each sample size. Each number in the x-axis is the sample size, i.e., the number of pedigrees simulated.

From 1000 replicates, we computed the bias for each model parameter. In Figure 3, the plots of these bias values are shown for all parameters in each model by sample size; the left is using the nuclear family data set and the right is with the extended pedigree data set. In each plot, the bars are grouped by three different parameter categories; coefficient parameter (denoted as *CO*), intercept parameter (denoted as *IN*) and variance component parameter (denoted as *VC*) within each sample size. In all models, the coefficient and intercept parameters were generally close to the true values while the variance component parameters tended to be overestimated. The direction of bias was preserved over varying sample sizes and data types for the each variance component parameter in Model 1, 2 and 4 while it was not in Model 3. With regard to sample size, the accuracy of parameter estimates increased with the bigger sample size as expected. The accuracy also increased with extended pedigree data, especially for the SEM analysis model (Model 3) and the CFA model (Model 4).

**Figure 4:**
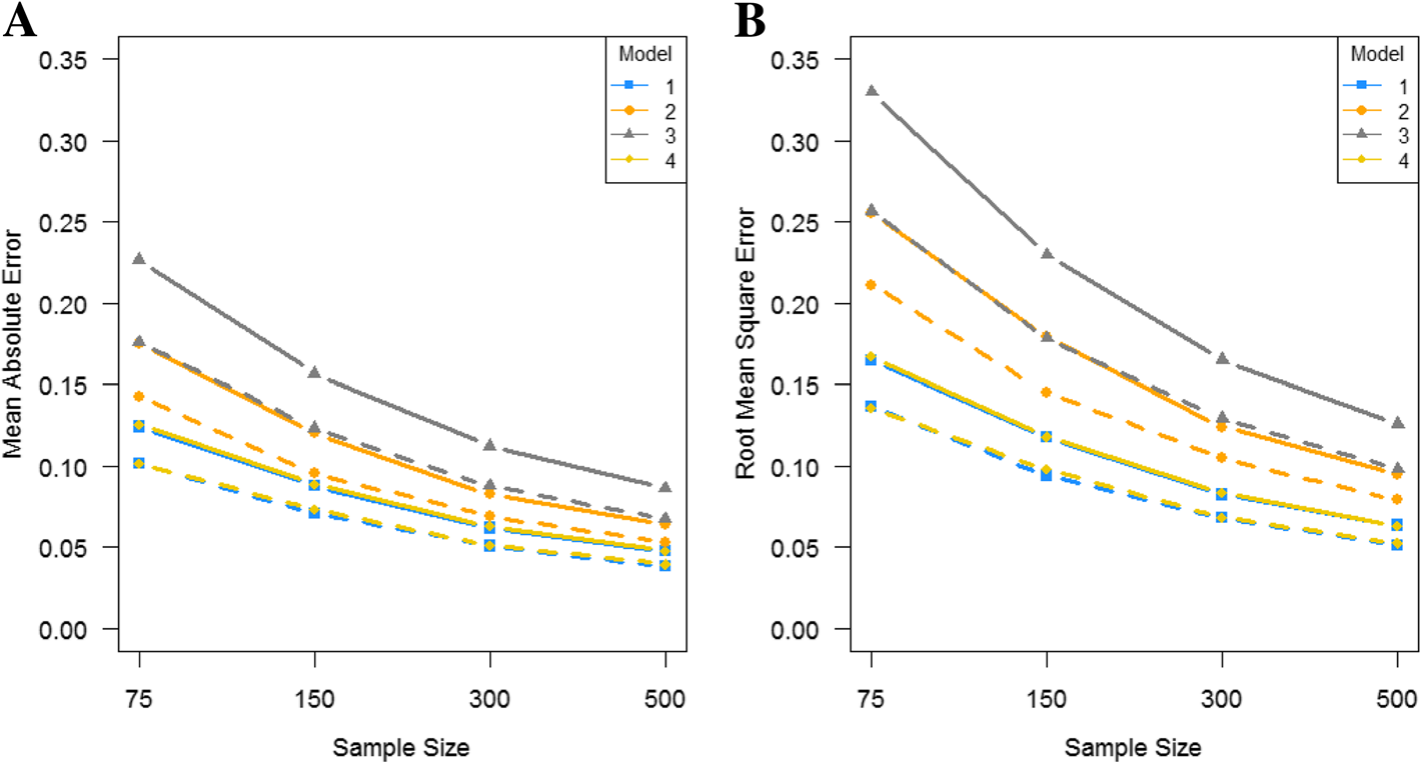
**Mean absolute deviation and root mean square error across all parameters values each model by sample size. A** is for mean absolute deviation, and **B** is for root mean square error. The solid lines are with the nuclear family data, and the dotted lines are with the extended pedigree data.

The mean of absolute deviation and the root mean squared error over all parameter values by sample size are shown in Figure 4 for each model and both data sets. The absolute deviations of parameter estimates decreased as the sample size was increased (4A). Among 4 analysis models, the SEM analysis model (Model 3) had the biggest absolute deviations for both data types. Overall, the absolute deviations were reduced with the extended pedigree data. The root mean squared error values had the same trend as the mean absolute error values (4B). It was bigger for the smaller sample size, especially the SEM analysis model (Model 3), but it decreased with the larger sample size.

### Coverage probability and type 1 error

**Figure 5:**
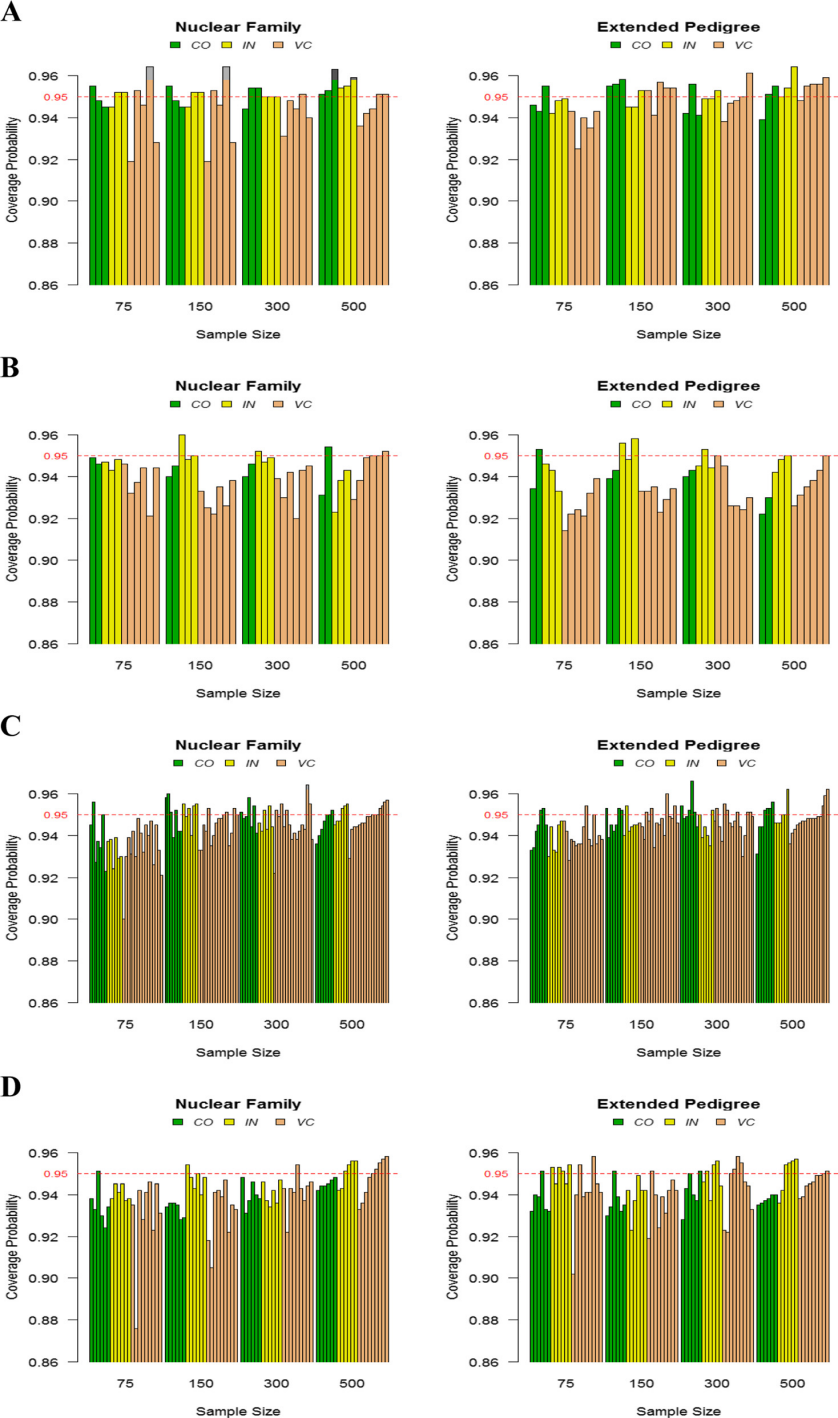
**The coverage probability of each parameter type by sample size. A** is for Model 1, **B** is for Model 2, **C** is for Model 3, and **D** is for Model 4 from Figure 1. Note that *CO* is for the coefficient parameters, *IN* is for the intercept parameters, and *VC* is for the variance component parameters of the models. The numbers in the x-axis in each plot represent the sample size, i.e., the number of pedigrees simulated.

Again for all parameters in each model and the sample size, the plots of the coverage probabilities for nominal 95% confidence interval are shown in Figure 5, by three different parameter categories within each sample size. All coverage rates of the 95% CI were very close to the nominal value for all sample sizes and both data types. No noticeable trends by the different parameter categories were observed for the coverage probabilities as observed in the bias estimates. The coverage probabilities for the linkage analysis model (Model 2) were a little lower than for other analysis models. The type 1 error rate for Model 1 is shown in Table 1. Overall, the type 1 error rate is a little inflated for the smaller sample size, but improves as the sample size get larger.

**Table 1:** **Type 1 error rate for analysis model 1**

	**Nuclear family**			**Extended pedigree**		
***α*** **level**	**0.05**	**0.01**	**0.001**	**0.05**	**0.01**	**0.001**
**Pedigree count**						
**75**	0.0582	0.0136	0.0015	0.0560	0.0114	0.0017
**150**	0.0545	0.0122	0.0013	0.0536	0.0116	0.0016
**300**	0.0513	0.0092	0.0009	0.0538	0.0115	0.0011
**500**	0.0500	0.0097	0.0012	0.0513	0.0097	0.0004

The results are from 10000 replicates.

### Test of model fit

The model fit statistic represents a test of the null hypothesis that the model used is correct versus the alternative hypothesis that the model is saturated [15]. The *strum* package reports the 4 different model fit measures with the degrees of freedom and p-values; the unadjusted chi-square index of fit, the mean adjusted chi-square index of fit, the mean and variance adjusted chi-square index of fit, and the theoretically corrected chi-square index of fit as described in the Implementation section.

**Figure 6:**
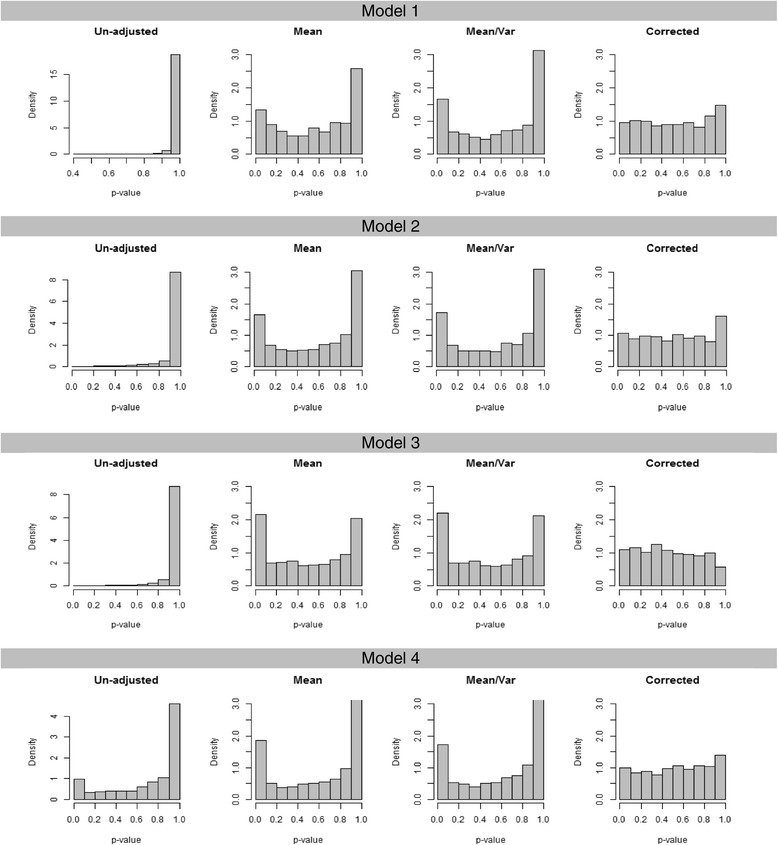
**Distribution of p-values of the test of model fit.**

In Figure 6, the histograms of p-values for the case with 75 extended pedigrees are shown for each analysis model. Though the distribution of p-values from the mean adjusted and the mean and variance adjusted tests of model fit were more uniform than the one from the unadjusted test, it still was distributed quite apart from the uniform distribution. Only the p-value from the theoretically corrected test was distributed close to the uniform distribution. Thus, we recommend that the theoretically corrected results be used.

### Ascertainment

**Figure 7:**
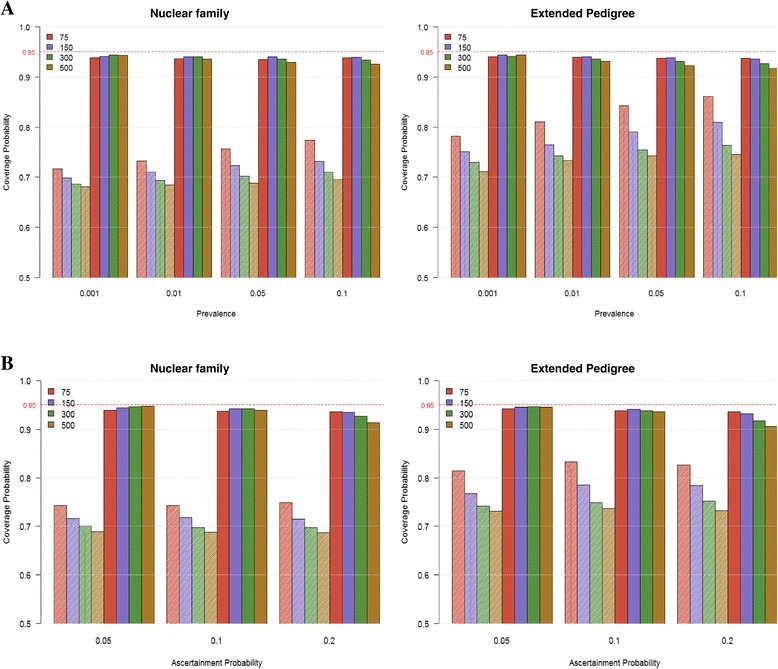
**The mean coverage probability of all parameters.** The coverage rate for each parameter in each scenario was estimated, and the average coverage rate over parameters and scenarios was calculated. **A** is by prevalence regardless of ascertainment probability, and **B** is by ascertainment probability regardless of prevalence. The shaded bars are without ascertainment correction, and the solid bars are with ascertainment correction. The number associated with a color in each plot represent the sample size, i.e., the number of pedigrees simulated.

The coverage probability of the ascertainment modeling in *strum* analysis is shown in Figure 7, compared to the result without the ascertainment correction. Each bar in the plot by prevalence shows the mean coverage probability from all ascertainment probabilities pooled for a sample size (7A), and vice versa for the bar by ascertainment probability (7B). Coverage rates for the different scenarios and parameters were averaged to get the overall picture, because the number of parameter and scenario combinations was large. As it is already known that the estimates are biased in the analysis with the ascertained pedigrees, the reflected coverage probabilities were far below the nominal value of 95% for all settings, regardless of sample size, prevalence and ascertainment probability (the shaded bars in Figure 7). Without ascertainment correction, for different sample sizes, the mean coverage probability of all parameters decreased as the sample size increased, since the pedigree likelihood would converge to the biased distribution. As the prevalence value increased, the mean coverage probability was improved slightly. Comparing to the results from the nuclear family data, the coverage rates were a little higher with the extended pedigree data.

On the other hand, the lost coverage probability was recaptured with the ascertainment modeling in *strum* analysis, very close to the nominal value of 95% for all settings and for both data types, again regardless of sample size, prevalence and ascertainment probability (the solid bars in Figure 7). We note that our ascertainment correction approach uses the single ascertainment approach which assumes that the ascertainment probability is very close to zero. Thus, as expected, for the biggest ascertainment value (0.2) we tested, the coverage probability showed a little decrease with the increased sample size for both data types (Figure 7B). The same pattern was observed with the larger prevalence values (0.1 and 0.05) for the extended pedigree data. As may be seen, the method is fairly robust to deviations from the “single ascertainment” assumption. However, if a very high proportion of the affected population is ascertained, our approach may not be adequate.

**Figure 8:**
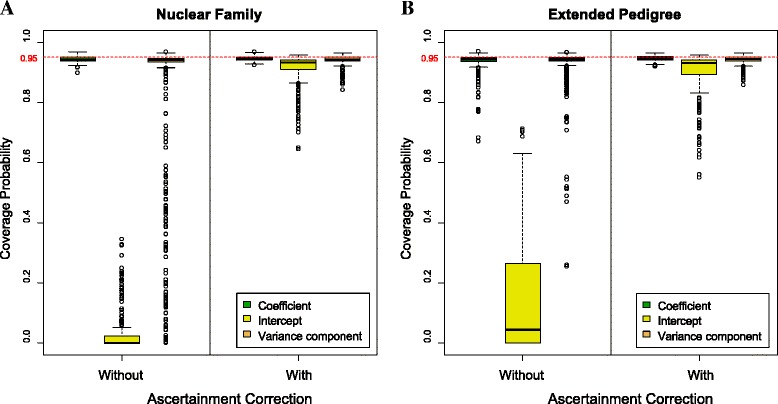
**The distribution of coverage probability by parameter type.** Each point in the graph represents the coverage rate for a specific parameter in a specific scenario. **A** shows coverage rates for the nuclear family data and **B** is for the extended pedigree data. In each plot, the left panel is without ascertainment correction, and the right panel is with ascertainment correction. Coverage rates for the different scenarios and parameters were collapsed together to get the overall picture, because the number of parameter and scenario combinations was large.

In Figure 8, the boxplots of the coverage probabilities, combined from all scenarios of prevalence, ascertainment probability and sample size, are shown for with and without ascertainment correction by parameter category; A is using the nuclear family data set and B is with the extended pedigree data set. For all parameter categories, some of the scenarios resulted in very poor coverage rates when ascertainment correction was not used. However, the intercept type parameters were particularly bad with a median coverage rate across all the parameters and settings of close to 0. As may be seen, regardless of the parameter category, many parameters had improved coverage in some of the investigated scenarios. The intercept showed an especially dramatic improvement. With ascertainment correction, the median coverage probabilities were close to the nominal value of 95%. Among three different parameter categories, the intercept parameter had the lowest coverage probabilities regardless of the data type, sample size, ascertainment probability, and prevalence. Although adjusting for ascertainment did not have a big effect on the median coverage probability across all the ‘Coefficient’ and ‘Variance Component’ parameters, coverage for many of these individual parameter was significantly improved. This may be seen by the fact that the number of low coverage outlier parameters is significantly smaller in the adjusted vs unadjusted graphs. These results show that our method for ascertainment correction can meaningfully improve the generalizability of the results obtained from ascertained pedigree data.

## Conclusions

Here, we present the package *strum,* which can be easily used with general pedigree data, giving it some significant advantages over other software packages. It improves upon existing SEM packages by allowing for multilevel modeling in general pedigrees. This flexibility allows the dissection of multiple influences on multifactorial traits, which is of key importance in genetic epidemiology. True pleiotropy occurs when common associations are observed between the same gene and unrelated phenotypes [31,32]. SEM can clarify this phenomenon by explicitly modeling correlation among related phenotypes, and potentially dispel distinctions between seemingly unrelated disorders. In addition, the flexibility to estimate polygenic variance allows the user to estimate heritability in the context of candidate gene effects and environmental influences. Also, to our knowledge, no other currently available SEM packages have a built-in approach for handling ascertainment. Our package has a helpful integrated tool for genetic data simulation. Finally, it has convenient built-in tools for model visualization. Therefore, the *strum* package provides a significant addition to biomedical research.

We presented the result from the simulation study for the performance evaluation of the new *strum* package. The parameter estimates were fairly unbiased with the proper coverage probabilities for all 4 models we tested. The p-value from the theoretically corrected test of model fit was properly distributed as uniform under the null hypothesis that the model used is correct, therefore we recommend to users to use the theoretically corrected test to if check the model fits the data. Also, the coverage probability with the ascertainment modeling in *strum* analysis was very close to the nominal value of 95% for all settings, while the result without the ascertainment correction showed the coverage probabilities were far below the nominal value.

Although our simulation study could not cover all possible scenarios that might be present in the real world, our results show that the *strum* package is very reliable and robust in terms of the accuracy and coverage of parameter estimates. Our package can be easily used with general pedigree data and it has robust support for modeling ascertainment, making it unique among SEM packages. Therefore, we conclude that *strum* is a valuable new tool for genetic analysis and it represents a step of progress for biomedical and genetic research.

### Ethics for the study

Ethical approval was not needed for this study because there were no human subjects data used and thus did not constitute human subjects research.

## Availability and requirements

**Project name**: strum

**Project home page**: http://cran.r-project.org/web/packages/strum/index.html

**Operating system**: Platform independent

**Programming languages**: R, C++

**Other requirements**: R (3.0.0 or newer); R-package: methods and pedigree from CRAN, Rgraphviz (≥2.6.0) from Bioconductor

**License**: GPL (≥ v2)

**Any restrictions to use by non-academics**: None

## Abbreviations

SEM: Structural equation modeling
CFI: Comparative fit index
CPU: Central processing unit
CFA: Confirmatory factor analysis
ACE: Additive genetic (A), shared environment (C), and residual environment (E)
CI: Confidence interval
IBD: Identity by descent
SNP: Single nucleotide polymorphism
SBP: Systolic blood pressure
DBP: Diastolic blood pressure
